# The Relationship between Mental and Somatic Practices and Wisdom

**DOI:** 10.1371/journal.pone.0149369

**Published:** 2016-02-18

**Authors:** Patrick B. Williams, Heather H. Mangelsdorf, Carly Kontra, Howard C. Nusbaum, Berthold Hoeckner

**Affiliations:** 1 Department of Psychology, The University of Chicago, Chicago, Illinois, United States of America; 2 Department of Music, The University of Chicago, Chicago, Illinois, United States of America; University of Udine, ITALY

## Abstract

In this study we sought to explore how experience with specific mental and somatic practices is associated with wisdom, using self-report measures of experience and wisdom. We administered standard surveys to measure wisdom and experience among four groups of practitioners of mental and somatic practices, namely, meditators, practitioners of the Alexander Technique, practitioners of the Feldenkrais Method, and classical ballet dancers. We additionally administered surveys of trait anxiety and empathy to all participants to explore possible mediating relationships of experience and wisdom by characteristics thought to be components of wisdom. Wisdom was higher on average among meditation practitioners, and lowest among ballet dancers, and this difference held when controlling for differences in age between practices, supporting the view that meditation is linked to wisdom and that ballet is not. However, we found that increased experience with meditation and ballet were both positively associated with wisdom, and that lowered trait anxiety mediated this positive association among meditation practitioners, and, non-significantly, among ballet dancers. These results suggest that not all practices that are purported to affect mental processing are related to wisdom to the same degree and different kinds of experience appear to relate to wisdom in different ways, suggesting different mechanisms that might underlie the development of wisdom with experience.

## Introduction

Although wisdom is considered the pinnacle of human cognition and has played a role in religion and philosophy reaching far back into human history, the scientific study of wisdom is a relatively recent phenomenon. As the study of wisdom has grown, a variety of ways to define the construct have emerged. Common themes include the skillful use of knowledge acquired through life experience, lowered anxiety in the face of difficult life decisions, careful reflection on the mental states of oneself and others, and action based in compassion and prosocial behavior [1]. The wisdom literature generally distinguishes between two types of wisdom: general wisdom, which represents insight into the pragmatics of life from a decentered third-person point of view, and personal wisdom, which an individual may acquire and cultivate through insight into daily life [2]. For the purposes of this study, we are interested in how individuals may cultivate personal wisdom through specific mental and somatic practices.

We conceptualize wisdom as a unified construct composed of interrelated cognitive, reflective, and affective characteristics [3]. In this model, wisdom is characterized as a deep and accurate perception of reality, in which insight into human nature and a diminished self-centeredness are acquired through life experience and practice in perspective taking. If wisdom exists as a set of cognitive, reflective, and affective characteristics, each susceptible to change over the lifespan, it is an open question whether experience with structured mental and somatic practices that cultivate these characteristics is associated with greater wisdom.

Mental and somatic practices may increase wisdom by providing what Glück and Bluck [4] refer to as positive general life resources, which affect the events an individual is likely to encounter in life, how such encounters are perceived and appraised, and how challenging experiences are integrated into a person’s life story. Experimental research into the malleability of wisdom suggests that wisdom is affected by training specific strategies for gaining knowledge, inferring insight from personal experience, and viewing difficult situations from a distanced perspective [5–8]. Certain structured mental and somatic practices that aim to affect these processes may therefore have positive effects on wisdom over time. In the current study, we provide a preliminary investigation into how specific types of life experiences may lead to the cultivation of wisdom and wisdom-related characteristics. We do this by surveying wisdom in individuals with varying levels of experience with four different mental and somatic practices: meditation, the Alexander Technique (AT), the Feldenkrais Method (FM), and classical ballet.

Meditation is a practice long associated with the development of wisdom in Buddhist and Taoist traditions [9,10]. Meditation may influence wisdom in multiple ways, for example by increasing interpersonal skills and by decreasing general anxiety through increased emotional self-regulation. Certain styles of meditation have been linked experimentally with increased compassion and prosocial behavior. For example, kindness-based meditation is associated with increased connectivity to and positive regard for others [11]. Highly experienced Buddhist meditators show greater self–other integration than non-religious controls [12]. Moreover, expert meditators (compared to novices) exhibit greater grey matter volume in regions of the brain associated with affective regulation [13], suggesting that increased social-connectedness, and subsequently increased compassion and prosocial behavior, are related to durable effects of meditation practice over time.

Wisdom is often characterized by the ability to face difficult situations with lowered stress and anxiety, and meditation may train the sort of emotional self-regulation that leads to this quiescent mental state. In experimental settings, brief meditation training has been associated with increased optimism and reduced recall of negative words [14,15], suggesting that meditation influences affect by reducing the impact of negative thoughts and stimuli. The results of a recent study indicate that, following brief meditation training, participants down-regulate thoughts emphasizing negativity but not those emphasizing positivity [16]. Though these studies used only brief meditation training with meditation-naïve participants, the results suggest that practicing emotional regulation in the course of meditation training leads to a decreased focus on negative thoughts and stimuli.

While the link between meditation practice and wisdom has been theorized since ancient times, somatic practices have been largely overlooked as potential means to foster wisdom. To our knowledge, no studies have examined whether physical practices of any kind are linked to the cultivation of personal wisdom, nor have they theorized that this association might exist. This may be due in part to the long-standing dualism that has dominated Western culture and scientific thought since Plato considered the human soul to be separate from the body and the body a distraction in intellectual life. Recent advances in embodied cognition, psychophysiology, and somatic therapies challenge these age-old conceptualizations and propose that our bodies have an important influence on the way we think and feel.

While embodied cognition has many different meanings (see Wilson,[17] for a review), they all hold that cognitive processes are deeply rooted in the body’s interactions with the world. One view is that thinking involves the reactivation of modality-specific brain areas involved in sensation and perception [18,19]. Even when we are not moving our bodies, our cognitive processes incorporate physical information from past experience. Since our bodies are an ever-present part of the context in which we use our minds, they influence the representations we form [20]. This view is supported by Kontra and colleagues [21], who found that specific bodily experiences bolster student learning of concepts in the physics classroom. Instructing children and adults to produce hand gestures has also been shown to enhance the learning of new material [22–26]. Additionally, movement patterns, such as changes in posture, can have a profound impact on mood and behavior. An open, expansive posture has been shown to increase feelings of power and tolerance of risk, decrease cortisol, and elevate testosterone levels [27]. Alternatively, hunched postures elicit more depressed feelings [28], and tilting the head upward induces pride [29]. To the extent that somatic training alters movement patterns (e.g., reinforces a more upright and open posture), this sort of bodily experience may contribute to psychological changes associated with wisdom.

While mental practices like meditation are often undertaken with the explicit goal of becoming more wise, somatic methods like AT and FM are typically practiced as a form of physical therapy, to achieve physical goals, or to enhance artistic performance. However, research suggests that these practices also have cognitive and emotional benefits related to components of wisdom. AT for example leads to increased emotional homeostasis [30], which may in turn lead to increased wisdom over time. The mind–body unity emphasized in AT greatly influenced philosopher John Dewey, who credited the practice with increased mental flexibility [31]. Research suggests that FM leads to both physical and mental improvements, including increased muscle length and flexibility [32], improvements in posture and muscle relaxation [33], reduced anxiety [34], as well as elevated mood, enhanced ability to learn, and increased clarity of thought [35]. Given the postulate that one’s mental state is reflected in and connected to the body, and based on the relative dearth of research into the connection between wisdom and somatic practices, we investigated the association between wisdom and experience with AT and FM.

In comparison to AT and FM, we also investigated classical ballet—a somatic practice that has also been associated with increased mental faculties. Since ballet has not been empirically linked with changes in many wisdom-related characteristics, we did not expect experience to be associated with personal wisdom. Research into the psychology of dancers suggests that the increased cognitive capabilities developed through ballet training are mainly limited to perception and memory for movements, though this limitation may be due to a lack of research into possible effects of dance experience on non-dance related cognition. Ballet dancers form and rely on increased body awareness to improve their physical performance [36], and experts are better able than novices to hold images of body-based movements in memory [37]. Furthermore, dancers show increased perceptual sensitivity to subtle changes in the movement of others [38]. As part of their training, dancers spend extensive periods of time observing their own and others’ bodies [39]. Ballet dancers also develop coping strategies to deal with performance anxiety and the pressures of professional success in the face of competition [40]. Given the nature of the changes observed in psychological studies of dance (i.e., they appear grounded in domain-specific cognition and affect), we did not hypothesize a link between ballet training and wisdom.

In the present study, we asked whether experience with mental and somatic practices is associated with wisdom. Previous research has connected wisdom with adverse life events, but we explore whether certain types of experience or training are associated with wisdom. We measured wisdom and several of its purported components with self-report questionnaires; these components included emotional self-regulation in the form of lowered trait anxiety as well as inter- and intrapersonal reactivity in the form of cognitive and affective empathy. We tested an hypothesized association between wisdom and experience with candidate mental and somatic practices—Meditation, Alexander Technique, and Feldenkrais Method, in comparison to another physical practice of classical ballet—including the mediating role of wisdom-related characteristics. By asking whether mental and somatic practices are associated with increased wisdom and exploring the relationship between the psychological construct of wisdom and its components, we aimed to lay the groundwork for a deeper understanding of the development of wisdom and to stimulate future work in the field. By examining both mental and somatic practices, it may be possible to identify aspects of life experiences that are relevant to the development of wisdom.

## Method

### Participants

Participants gave consent to participate in the study by agreeing to complete the online survey. This study was approved for online administration by the University of Chicago Institutional Review Board (IRB12-1920). Participants were recruited via online solicitations to respective companies and schools across the United States who had email addresses available online, as well as through email list-servs at the University of Chicago. Participants gave consent by agreeing to complete the online survey. Each participant was compensated with a $3 online gift card, with a chance to win a $100 online gift card at the conclusion of the study. Across all practice types, 452 individuals responded to the email and other solicitations. Of these 303 completed the survey in full, and an additional 6 participants were removed because their reported years of experience was greater than 2.5 standard deviations from the mean of their respective group, leaving 298 participants in the final set of analyses.

Participants from the AT sample ranged in age from 24 to 80 (mean = 50.49, sd = 13.76), Ballet dancers ranged in age from 11 to 62 (mean = 25.35, sd = 7.37), FM participants ranged in age from 27 to 76 (mean = 52.8, sd = 11.68), and meditation participants ranged in age from 18 to 68 (mean = 50.49, sd = 13.76). See Table 1 for a breakdown of group level descriptive statistics.

**Table 1:** Means and standard deviations of wisdom, its components, and predictor variables.

	AT		Ballet		FM		Meditation	
	M	SD	M	SD	M	SD	M	SD
Tot wisdom	3.90	.33	3.59	.37	3.92	.39	4.01	.36
Cog Wisdom	4.25	.45	4.00	.57	4.20	.58	4.30	.35
Refl Wisdom	3.66	.36	3.31	.47	3.83	.47	3.88	.46
Aff Wisdom	3.79	.47	3.47	.45	3.74	.44	3.85	.53
Years	19	12.07	13.44	9.69	14.05	8.74	9.56	7.81
Age	50.49	13.76	24.74	6.02	52.80	11.68	41.61	14.55
TA	1.81	.41	2.18	.48	1.84	.46	1.88	.46
CE	3.22	.41	3.09	.40	3.17	.40	3.13	.41
AE	2.42	.24	2.50	.21	2.41	.27	2.46	.25

### Materials and procedure

The survey was administered via the SurveyMonkey website. Participants first gave consent and then read a statement indicating the purpose of the survey—to gather information about experience with and possible benefits of a particular practice. The experience section of the survey included questions regarding experience as a student and/or teacher of meditation, AT, FM, or ballet. Experience with each practice was operationalized as the self-reported number of years of practice (continuous), as well as estimated hours of practice (categorical). The psychological questionnaires included the Three-Dimensional Wisdom Scale [41], the Questionnaire for Cognitive and Affective Empathy (QCAE [42]), and the Trait Anxiety portion of the State-Trait Anxiety Inventory for Adults (STAI-Y [43]). Participants lastly completed a demographic questionnaire that asked for information on age, income, and gender.

#### Measures

*Three-Dimensional Wisdom Scale*. The 3D-WS is a measure of cognitive, reflective, and affective dimensions of wisdom [41,44]. The 3D-WS has adequate construct, content, predictive, discriminant, and convergent validity, among older populations [45] and is a valid measure of wisdom among young adults of varying cultural backgrounds [46–49]. Examples from the individual subscales include: cognitive dimension—*A person either knows the answer to a question or he/she doesn’t (reverse scored)*; reflective dimension—*I try to look at everybody’s side of a disagreement before I make a decision*; and affective dimension—*It’s not really my problem if others are in trouble and need help (reverse scored)*.

*Questionnaire for Cognitive and Affective Empathy*. The QCAE [42] measures cognitive empathy using the *Perspective Taking* and *Online Simulation* subscales, and affective empathy using the *Emotional Contagion*, *Proximal Responsivity*, and *Peripheral Responsivity* subscales. *Perspective Taking* refers to the ability to take on the perspective of another person, while *Online Simulation* involves imagining what another is feeling. *Emotional Contagion* is a measure of the automatic mirroring of another’s emotions. *Proximal Responsivity* is ones’ response when witnessing the mood of someone else in a close social context, while *Peripheral Responsivity* refers to the same type of response but in a detached social context. The cognitive and affective portions of the QCAE show convergence with the Basic Empathy Scale (BES [50]). For this study, four items that overlap with the 3D-WS were removed from the *Online Simulation* subscale of cognitive empathy, leaving 5 of the original 9 items.

*State-Trait Anxiety Inventory—Trait Anxiety*. The Trait Anxiety portion of the STAI Form Y contains 20 items related to typical daily experiences of stress, discomfort and worry. Respondents rate each item (e.g., “I worry too much over something that doesn’t really matter”) on a 4-point Likert-type scale, ranging from 1 (almost never) to 4 (almost always). Considerable evidence attests to the construct and concurrent validity of the scale [43,51]. Internal consistency coefficients for the scale have ranged from .86 to .95 and test-retest reliability coefficients have ranged from .65 to .75 over a 2-month interval [43].

#### Data Analyses

Analyses of Variance (ANOVAs) were performed to investigate the differences between groups in wisdom, cognitive empathy, affective empathy, and anxiety, followed by a similar analysis after partialling out variance due to individual differences in age. Ordinary least squares (OLS) regression models were constructed to test whether experience with each practice is associated with individual differences in wisdom, controlling for covariates age, gender, and income. Though we used years of experience as the dependent measure, in a separate analysis OLS models were constructed using either years of experience (continuous, self-report, ranging from 1 to 44 years) or hours of experience (categorical, self-report, ranging from 0–5 hours to greater than 40,000 hours) to ensure that using one or the other dependent measure did not significantly alter the pattern of results. For groups in which significant variance in wisdom was associated with experience, steps for determining mediation were performed separately for cognitive empathy, affective empathy, and trait anxiety as mediators [52], after partialling out covariance from age, gender, and income. Mediation analyses were tested for significance using the Sobel test for standard error [53].

## Results

### Wisdom and wisdom related characteristics between groups

An ANOVA revealed no significant differences between groups for measures of cognitive empathy (CE) or affective empathy (AE), and a significant difference between groups in trait anxiety (TA; *F*(3,290) = 11.06, *p* < .001). Posthoc analyses using Tukey's HSD test revealed significantly higher TA among ballet dancers (*M* = 2.18, *SD* = 0.48) than other groups combined (*M* = 1.84, *SD* = 0.44, *p* < .001). There were no significant differences in average TA between AT, FM, or meditation practitioners. An ANOVA comparing practice types on income indicated that ballet dancers are in a lower income level than other practices (ballet: $35,000 –$49,999, other practices: $50,000–74,999, p < .05).

An ANOVA with wisdom as the dependent variable and practice type (regardless of amount of practice) as the quasi-independent variable revealed significant differences in wisdom between groups (*F*(3,290) = 18.49, *p* < .001). Posthoc analysis using Tukeys HSD test revealed that ballet dancers reported significantly lower average wisdom scores (*M* = 3.59, *SD* = 0.37) than the other groups combined (*M* = 3.94, *SD* = 0.36, *p* < .001). There were no significant differences in average wisdom scores between AT, FM, or meditation practitioners. Because ballet dancers were much younger than other groups (ballet mean = 24.74, other practices mean = 49.26, t = 21.15, p < .001), and because age is traditionally associated with differences in wisdom, this analysis was repeated after variation accounted for by differences in age were statistically removed from the dependent variable wisdom. An ANOVA with the residual of wisdom (after statistically removing variation due to age) as the dependent variable and practice type as the quasi-independent variable revealed significant differences in wisdom between groups (*F*(3,290) = 7.13, *p* < .001) with meditation having the largest and ballet the smallest residual wisdom score. Posthoc analyses using Tukeys HSD indicate that significant differences occurred only between meditation (*resid*. *M* = .17, *SD* = .38) and FM (*resid*. *M* = .004, *SD* = .39, p < .05), between meditation and ballet (*resid M*. = -0.13, *SD* = .37, p < .001), and a trending difference between meditation and AT (resid. M = .004, SD = .32, p = .06).

### The association between wisdom and practice

Two sets of ordinary least squares (OLS) regression models were constructed to investigate the association between wisdom and experience with mental and somatic practices, controlling for covariation in age, income, and gender, using either self-reported years of experience or self-reported hours of experience. See Table 2 for a breakdown of average years of experience and frequency counts of hours of experience across practice types. For all practice groups, there was not a significant difference in the models that use years of experience compared to the models that use hours of experience. Because years of experience had a higher level of granularity—ranging from a single year up to 44 years (in the case of the Alexander Technique)—we chose to use this measure over hours of experience—where participants chose from eight categories ranging from less than five hours, up to greater than 40,000 hours.

**Table 2:** Experience across practice types.

Hours	N	AT	Ballet	FM	Meditation
Average years experience^a^	-	19.0	13.4	14.1	9.6
Fewer than 5 hours^b^	10	0	6	1	3
6–10 hours	1	0	0	0	1
11–100 hours	14	3	4	2	5
101–1,000 hours	64	6	23	17	18
1,001–10,000 hours	107	34	14	40	19
10,001–20,000 hours	47	14	16	13	4
20,001–40,000 hours	21	6	5	8	2
Greater than 40,000 hours	31	2	14	13	2

^a^Average years taken from self-reported years of experience.

^b^Frequency of hours taken from 8-level categorical self-reported experience.

The association between meditation practice and wisdom was significant (*R*^2^ *(adj)* = 0.28, *F*(9,44) = 3.3, *p* < .01), as was the association between wisdom and experience with ballet dance (*R*^2^ *(adj)* = 0.24, *F*(9,71) = 3.8, *p* < .001), controlling for age, gender, and income. There was no significant association between AT experience and wisdom (*R*^2^ *(adj)* = -0.01, *F*(9,55) = 0.96, *n*.*s*.), nor was there a significant association between FM experience and wisdom (*R*^2^ *(adj)* = -0.03, *F*(9,84) = 0.72, *n*.*s*.), controlling for age, gender, and income. For a full description of the simple correlations between experience with each practice and wisdom, it’s subcomponents, and individual characteristics, see Table 3.

**Table 3:** Correlations among wisdom, its subcomponents, and predictor variables.

Variables	1	2	3	4	5	6	7	8	9
Alexander Technique (N = 65, 56 females)									
1. Tot wis	-								
2. Cog wis	.77***	-							
3. Refl wis	.68***	.25*	-						
4. Aff wis	.83***	.45***	.40***	-					
5. Years	.05	-.03	-.05	.17	-				
6. Age	.22	.21	.08	.19	.58***	-			
7. TA	-.49***	-.33**	-.49***	-.32**	-.14	-.25*	-		
8. CE	.44***	.23	.41***	.39**	-.12	-.06	-.20	-	
9. AE	.40**	-.25*	-.41***	-.28*	.07	-.11	.25*	-.27*	-
Ballet (N = 81, 76 females)									
1. Tot wis	-								
2. Cog wis	.82***	-							
3. Refl wis	.64***	.22*	-						
4. Aff wis	.78***	.54***	.26*	-					
5. Years	.45***	.42***	.20	.38***	-				
6. Age	.11	.18	-.07	.13	.46***	-			
7. TA	-.31**	-.32**	-.16	-.20	-.31**	-.11	-		
8. CE	.30**	.26*	.14	.27*	.05	.16	-.10	-	
9. AE	.08	.13	-.09	.12	-.01	.10	.09	.12	-
Feldenkrais Method (N = 94, 72 females)									
1. Tot wis	-								
2. Cog wis	.84***	-							
3. Refl wis	.74***	.41***	-						
4. Aff wis	.76***	.48***	.36***	-					
5. Years	.14	.15	.14	.02	-				
6. Age	.04	.16	.09	-.18	.41***	-			
7. TA	-.33**	-.20*	-.35***	-.24*	-.04	-.10	-		
8. CE	.51***	.39***	.42***	.39***	.11	-.04	-.15	-	
9. AE	.06	.14	-.02	-.01	-.05	.04	27**	-.26*	-
Meditation (N = 54, 29 females)									
1. Tot wis	-								
2. Cog wis	.75***	-							
3. Refl wis	.80***	.42*	-						
4. Aff wis	.86***	.52***	.49***	-					
5. Years	.33**	.27*	.34*	.21	-				
6. Age	-.01	.02	-.03	-.02	.62***	-			
7. TA	-.50***	-.29*	-.57***	-.34**	-.52***	-.27*	-		
8. CE	.53***	.35**	.59***	.35**	.17	-.04	-.24	-	
9. AE	-.20	.00	-.27*	-.17	-.18	-.15	.22	-.23	-

* p < .05,

** p < .01,

*** p < .001.

Meditation styles were heterogeneous across respondents, with most reporting multiple styles to the open-ended question *what style(s) of meditation do you have experience with*? Across the meditation sample 29.49% of respondents reported practicing *vipassana*, 23.08% reported practicing *mindfulness*, and 14.10% reported practicing *Buddhist* meditation. Table 4 displays the top ten styles by frequency and percentage of use in the meditation sample. Meditation styles are not mutually exclusive, and so percentages add up to more than 100.

**Table 4:** Frequency of mediation practice type.

Meditation type^a^	Frequency	Percentage
Vipassana	23	29.49
Mindfulness	18	23.08
Buddhist	11	14.10
Tibetan	10	12.82
Zen	9	11.54
Loving-Kindness	8	10.26
Mantra	7	8.97
Insight	6	7.69
Moving	6	7.69
Shamatha	6	7.69

^a^Meditation types overlap within participants and so percentages do not add up to 100.

### Mediation of the relationship between practice and wisdom

To investigate possible mediating effects of wisdom related characteristics on the relationship between practice and wisdom, OLS regression models were created in three steps. For these steps, as well as for Sobel tests, the covariates age, gender, and income were partialled out of the dependent variable *wisdom*. First the direct effect of the predictor variable *experience* was tested against the dependent variable *wisdom*. Secondly, each hypothesized mediating variable *CE*, *AE*, or *TA* was regressed against the dependent variable. Finally the predictor variable was regressed on the dependent variable, controlling for each mediating variable (in separate models). If when controlling for a mediator, the direct effect of the predictor on the dependent variable is no longer significant, then mediation may be assumed. This mediation was tested for statistical significance using the Sobel test of indirect effects, also known as the Sobel test for standard error [53].

Initial OLS analyses revealed a statistically significant positive association between meditation experience and wisdom (β = 0.34, *p* = 0.01). Among meditation practitioners, additional analyses revealed a significant negative association between experience and TA (β = -0.52, *p* < .001), though meditation experience was not significantly associated with CE or AE. Additional analyses revealed a significant negative association between TA and wisdom (β = -0.44, *p* < .001), indicating a potential mediating relationship of TA in the association between meditation experience and wisdom. There was a significant positive association between CE and wisdom (β = 0.45, *p* < .001) for people practicing meditation, and no significant association between AE and wisdom. Further analysis indicated that when TA is included in the model regressing meditation experience on wisdom, the relationship between practice and wisdom is no longer significant (β = 0.15, *p* = 0.3). Sobel's test revealed that this mediating effect is significant (*p* < .05).

As indicated by previous analyses, OLS regression revealed a significant positive association between ballet experience and wisdom (β = 0.38, *p* < .001). Further analyses among ballet dancers revealed a significant negative association between experience and TA (β = -0.31, *p* < .001), while experience with ballet dance was not significantly associated with CE or AE. In our ballet sample, there was a significant negative association between TA and wisdom (β = -0.24, *p* = 0.03), a significant positive association between CE and wisdom (β = 0.23, *p* = 0.04), and no significant relationship between AE and wisdom. When TA was included in a model regressing ballet experience on wisdom, the relationship between experience and wisdom decreased in magnitude (β = 0.33, *p* < .01), and Sobel's test of the indirect effect was not significant (*p* = 0.25), indicating that the relationship between ballet training and wisdom may be only partially mediated by TA.

The mean age of ballet dancers was significantly lower than for the three other practice groups (ballet mean = 24.74, sd = 6.02, other mean = 49.26, sd = 13.82; t(286.97) = 21.15, p < .001). To further investigate the association between experience and wisdom among ballet dancers and to see if experience and wisdom associations may have been driven by very experienced older dancers, we split the ballet group at the median of age and ran OLS analyses separately on the low and high age groups, with wisdom as the dependent variable and years of experience as the predictor, controlling for income and gender. Though the relationship between experience and wisdom was significant in both the lower and upper age groups (lower quantile: β = 0.59, *p* < .001; upper quantile: β = 0.33, *p* = .05), the association was stronger among the lower age group than the upper. Trait anxiety was significantly and negatively associated with wisdom among the older quantile (β = -0.36, *p* < .05), but not the lower age quantile (β = -0.22, *n*.*s*.), and it did not mediate the relationship between experience and wisdom in either group, mirroring the results in the group as a whole.

## Discussion

The study explored the relationship between mental and somatic practices and wisdom by measuring self-reported wisdom and several of its purported components to test an hypothesized association between wisdom and experience with candidate mental and somatic practices. We found that on average, controlling for differences in age, meditation practitioners reported higher wisdom than practitioners of the Alexander Technique (AT), the Feldenkrais Method (FM), or ballet dancers, with ballet dancers showing the lowest average levels of reported wisdom. Additionally, regression analyses revealed that wisdom is positively associated with years of experience in both meditation and ballet, but not with years of experience with AT or FM. Finally, the association between experience and wisdom is completely mediated by lowered trait anxiety among meditators, though this mediating relationship is not significant among ballet dancers. Taken together, these results suggest first that the practice of meditation is related to increased wisdom, and that this relationship may be due to an effect of prolonged meditation on lowered everyday anxiety. Second, though not associated with heightened wisdom *on average*, prolonged ballet experience may be linked to increased wisdom, though the current study does not indicate any potential causal mechanism for this association. As a caveat, the relationship between experience and ballet appears to be driven by the participants in the lower ranges of experience, suggesting that ballet may increase the resources needed to develop wisdom in early years of practice.

Before discussing our findings, we must first acknowledge the limitations of our self-report questionnaire and our cross-sectional design. Researchers lack a consensus on the best way to define and measure personal wisdom and while some behavioral measures exist [54,55], their administration and coding is time-intensive, and their operationalization of wisdom differs from the definition we chose for the present study. Despite the strong construct, content, predictive, discriminant, and convergent validity of the 3D-WS [45], the scale has the typical limitations of self-report methodology, including differences in participants’ introspective ability, interpretation of rating scales, potential dishonesty, demand characteristics, and desire for image management. Another concern with assessing wisdom through self-report is that wise individuals might temper their responses due to their humility, even though their increased reflective ability and self-knowledge should counteract their desire to provide humble responses (cf. Tiberius [56]). However, it is interesting that our sample of ballet dancers reported significantly lower wisdom on average than meditators, after partialling out effects of age between groups. Future research is needed to see if this finding can be replicated, or if it was due to demographic or other differences between our ballet and other practice samples.

The cross-sectional nature of our survey study, compared to longitudinal alternatives, prevents us from making any causal claims about meditation or ballet experience leading to increases in wisdom. It is possible that wise individuals are more likely to stick with a given practice over time. However, such a view of perseverance or grit [57] should predict that all four groups show the same association between years of practice and wisdom. However, while ballet dancers scored significantly lower on wisdom than meditators and people drawn to AT and FM, both the meditators and ballet dancers show systematic changes in wisdom with years of practice, and this is not seen for the other two groups. Longitudinal intervention studies, in which people with no prior meditation or ballet experience receive training over time, will be needed to determine if differences in the difficulty and nature of these practices leads to the development of wisdom and over what time frame.

Despite these limitations, the present results provide the first demonstration of an association between mental and somatic practices and wisdom, and they suggest that trait anxiety might mediate this relationship in some circumstances. While past research has shown that mindfulness meditation and dance training can lead to decreases in trait anxiety [58–61], no prior research has explored this outcome in connection to wisdom. In alignment with past research, our results suggest a negative relationship between trait anxiety and both meditation and ballet experience. Furthermore trait anxiety mediated the association between meditation practice and wisdom in our sample, and partially mediated the relationship between ballet practice and wisdom, suggesting one way in which meditation and wisdom may be linked. The negative relationship between trait anxiety and wisdom was seen only with the younger half of the surveyed ballet dancers, so that the change in the magnitude of the relationship with age may help explain the difference in associations between meditation, ballet, wisdom, and anxiety. Although we cannot determine causality from our data, the negative relationship between trait anxiety and wisdom suggests the possibility that meditation and ballet training may contribute to wisdom by training a person to avoid, manage, or overcome personal anxiety or anxieties inherent in each practice and in life in general.

Our findings suggest that training in meditation and ballet may relate to wisdom through helping people develop wisdom related resources, such as resilience to adversity. The ability to deal successfully with hardship correlates with an increase in psychological health for elders identified as wise and may be a prerequisite for the development of wisdom [3]. Meditation practice in general, and mindfulness practice in particular, is associated with improvements in psychological well-being as evidenced by improvements across a variety of psychiatric disorders, such as depression, anxiety, and addiction [62]. By improving psychological health, it is possible that practicing meditation helps people to deal with hardship in a more successful and wise manner by arming them with the resources needed to handle the challenge rather than viewing it as a threat. To the degree that practicing ballet improves psychological health and requires persistence, dancing could similarly help people cope with hardship, particularly in early stages of practice, even though most people do not begin ballet training with this purpose in mind.

In identifying relationships between mental and somatic practices and wisdom, the present study suggests several potential avenues for future research. Training studies should determine if meditation and ballet directly contribute to the development of wisdom and should examine additional wisdom-related characteristics developed through mental and somatic practices that might mediate the link between practice and wisdom. In addition, our findings should be replicated with multiple measures of wisdom to see if the relationships hold up with behavioral or other self-report assessments (see Glück et al [4]). If mental and somatic practices can indeed help cultivate wisdom, their applications should be explored across settings such as in the classroom or workplace with the goal of creating not only wiser people but also a wiser society that may benefit future generations.

## Supporting Information

S1 File Minimal data file. Comma separated value (csv) file from which all data analyses were performed.
(CSV)
